# Current recommendations for cancer surveillance in Gorlin syndrome: a report from the SIOPE host genome working group (SIOPE HGWG)

**DOI:** 10.1007/s10689-021-00247-z

**Published:** 2021-04-16

**Authors:** L. Guerrini-Rousseau, M. J. Smith, C. P. Kratz, B. Doergeloh, S. Hirsch, S. M. J. Hopman, M. Jorgensen, M. Kuhlen, O. Michaeli, T. Milde, V. Ridola, A. Russo, H. Salvador, N. Waespe, B. Claret, L. Brugieres, D. G. Evans

**Affiliations:** 1 Gustave Roussy Cancer Center, Department of Pediatric and Adolescent Oncology, Paris-Saclay University, Villejuif, France; 2 « Génomique Et Oncogénèse Des Tumeurs Cérébrales Pédiatriques » INSERM U981, Gustave Roussy Cancer Center and Paris-Saclay University, Villejuif, France; 3 Manchester Centre for Genomic Medicine, St Mary’s Hospital, Manchester Academic Health Science Centre, School of Biological Sciences, Division of Evolution and Genomic Science, University of Manchester, Manchester, M13 9WL UK; 4 Department of Pediatric Hematology and Oncology, Hannover Medical School, Hannover, Germany; 5 Institute of Human Genetics, Heidelberg University Hospital, Heidelberg, Germany; 6 Hopp Children’s Cancer Center Heidelberg (KiTZ), Heidelberg, Germany; 7 Department of Genetics, University Medical Center Utrecht, Utrecht, The Netherlands; 8 MJ Great Ormond Street Hospital for Children NHS Foundation Trust, London, WC1N 3JH UK; 9 Paediatric and Adolescent Medicine, University Medical Center Augsburg, Augsburg, Germany; 10 Division of Hematology/Oncology, Schneider Children’s Medical Center of Israel, Petah Tikva, Israel; 11 Department of Pediatric Hematology and Oncology, MITERA Children’s Hospital, Athens, Greece; 12 Department of Pediatric Hematology/Oncology, Center for Pediatric and Adolescent Medicine, University Medical Center of the Johannes Gutenberg-University Mainz, 55131 Mainz, Germany; 13 Pediatric Oncology Department, Sant Joan de Deu Children’s Hospital, Barcelona, Spain; 14 Institute of Social and Preventive Medicine, University of Bern, Bern, Switzerland; 15 Research Platform for Pediatric Oncology and Hematology, Faculty of Medicine, University of Geneva, Geneva, Switzerland; 16 Gustave Roussy Cancer Center, Psycho-oncology Unit, Paris-Saclay University, Villejuif, France

**Keywords:** Gorlin syndrome, *SUFU*, *PTCH1*, Surveillance, Hereditary, Cancer predisposition syndrome

## Abstract

**Gorlin syndrome (MIM 109,400), a cancer predisposition syndrome related to a constitutional pathogenic** variation (PV) of a gene in the Sonic Hedgehog pathway (*PTCH1* or *SUFU)*, is associated with a broad spectrum of benign and malignant tumors. Basal cell carcinomas (BCC), odontogenic keratocysts and medulloblastomas are the main tumor types encountered, but meningiomas, ovarian or cardiac fibromas and sarcomas have also been described. The clinical features and tumor risks are different depending on the causative gene. Due to the rarity of this condition, there is little data on phenotype-genotype correlations. This report summarizes genotype-based recommendations for screening patients with *PTCH1* and *SUFU*-related Gorlin syndrome, discussed during a workshop of the Host Genome Working Group of the European branch of the International Society of Pediatric Oncology (SIOPE HGWG) held in January 2020. In order to allow early detection of BCC, dermatologic examination should start at age 10 in *PTCH1*, and at age 20 in *SUFU* PV carriers. Odontogenic keratocyst screening, based on odontologic examination, should begin at age 2 with annual orthopantogram beginning around age 8 for *PTCH1* PV carriers only. For medulloblastomas, repeated brain MRI from birth to 5 years should be proposed for *SUFU* PV carriers only. Brain MRI for meningiomas and pelvic ultrasound for ovarian fibromas should be offered to both *PTCH1* and *SUFU* PV carriers. Follow-up of patients treated with radiotherapy should be prolonged and thorough because of the risk of secondary malignancies. Prospective evaluation of evidence of the effectiveness of these surveillance recommendations is required.

## Introduction

Gorlin syndrome (GS) (MIM 109,400), also called nevoid basal cell carcinoma syndrome (NBCCS) or basal cell nevus syndrome (BCNS), is an autosomal dominant inherited syndrome characterized by diverse developmental defects including macrocephaly, hypertelorism, skeletal anomalies, palmar and/or plantar pitting and a predisposition to various tumors. These can be malignant, such as basal cell carcinomas (BCC) and medulloblastoma, or benign such as keratocystic odontogenic tumors and ovarian or cardiac fibromas.

Due to the rarity of the syndrome and the variability of clinical signs, diagnosis of GS may be difficult [1]. These factors led to the definition of clinical diagnostic criteria using a combination of major and minor criteria on which several groups have agreed (Table 1).

**Table 1:** Clinical diagnostic criteria for Gorlin syndrome

Major criteria	Minor criteria
(1) BCC prior to 20 years of age or excessive numbers of BCCs (> 5 BCC) out of proportion to prior sun exposure and skin type (2) Odontogenic keratocyst of the jaw prior to 20 years of age (3) Palmar or plantar pitting (4) Lamellar calcification of the falx cerebri; (5) Medulloblastoma, typically desmoplastic (6) First degree relative with BCNS	(1) Rib anomalies; bifid/splayed/extra ribs (2) Other specific skeletal malformations and radiologic changes (i.e., vertebral anomalies, kyphoscoliosis, short fourth metacarpals, postaxial polydactyly) (3) Macrocephaly; (OFC > 97th centile) (4) Cleft lip/palate (5) Ovarian/cardiac fibroma (6) Lymphomesenteric or pleural? cysts (7) Ocular abnormalities (i.e., strabism, hypertelorism, congenital cataracts, glaucoma, coloboma)

Diagnosis of Gorlin syndrome requires

Two major diagnostic criteria and one minor diagnostic criterion or one major and three minor diagnostic criteria

Identification of a heterozygous germline *PTCH1* or *SUFU* pathogenic variant on molecular genetic testing

The genetic basis of the syndrome is the presence of a constitutional alteration in a gene within the Sonic Hedgehog pathway. Most GS cases are associated with heterozygous *PTCH1* pathogenic or likely pathogenic variants (PV). More recently, germline variants in *SUFU* [2] and rarely *PTCH2* [3] genes have also been described in patients with a Gorlin phenotype. Due to the rarity of this condition, there is little data on phenotype-genotype correlations [4].

Recently, the Host Genome Working Group (HGWG) was established in the European branch of the International Society of Pediatric Oncology (SIOPE) with the aim of improving care for patients with pediatric cancer predisposition syndromes. One of the issues addressed during the first workshop of this group, held in January 2020, was the development of surveillance guidelines in several predisposition syndromes. Several experts in the field of pediatric oncogenetics, including clinical geneticists and pediatric oncologists from European countries, participated in the meeting. Based on the current data, the experts’ recommendations and the consensus reached, the SIOPE HGWG now proposes a genotype-based surveillance program for patients with GS, but this is not yet fully evidence based. This report summaries the discussions and expert recommendations of this working group.

## Genetic associations

### Population incidence for *PTCH1* and *SUFU* related Gorlin syndrome

GS is suspected on phenotypic criteria and clinical diagnosis is established in a patient with two major diagnostic criteria and one minor diagnostic criterion or one major and three minor diagnostic criteria. (Table 1) [1, 5]. This can be molecularly confirmed by genetic analysis of the *PTCH1*, *PTCH2* and *SUFU* genes. All features of GS except odontogenic keratocysts [4, 6] can be present in *SUFU*-related GS, but GS clinical features are less prominent in people with germline *SUFU* PV and many will not meet GS criteria even in later life [6]. Current testing in Manchester of patients meeting GS clinical criteria show that 134/193 (69.4%) patients have an identifiable germline *PTCH1* PV, 11/193 (5.7%) have an identifiable *SUFU* PV and 48 (24.9%) have no identifiable PV in *SUFU, PTCH1* or *PTCH2*. No PV of *PTCH2* has been identified in this series, thus this gene is probably rarely involved in GS pathogenesis and was therefore not taken into consideration in these recommendations. The estimated birth incidence of clinical GS is 1 in 14,963 [7], suggesting that most of those with germline *PTCH1* PVs (estimated frequency 1 in 3356-gnomAD; https://gnomad.broadinstitute.org/) may never reach a clinical diagnosis. As the clinical signs of GS are even less prominent in patients with germline *SUFU* variant, a large number of *SUFU* PV carriers probably remain undiagnosed.

### Tumor risk

Most patients with GS develop several benign and malignant tumors, most often at a young age. There are some differences in the clinical features and incidence of tumor occurrence depending on the gene involved (Table 2). Even though it is dominated by BCC and keratocystic odontogenic tumors, the spectrum of tumors associated with GS is large. Ovarian fibromas, meningiomas and medulloblastomas appear to be more frequent in patients with constitutional pathogenic *SUFU* variants than *PTCH1* variants [4].

**Table 2:** Tumor risk in GS patients: main series from the literature

	Shanley [22] Australia	Kimosis [9] USA	Endo [10] Japan	Evans [4] UK		
Number of patients	118	105	157	182		
Variant identified				*PTCH* 126	*SUFU* 9	None 47
Median age at last follow-up	35	34.5	33.1	45	42	47
BCC*	90 (75%)	71 (80%)	56 (37.8%)	61 (48%)	4 (44%)	21 (45%)
KCOT	85/113 (75%)	78 (81%)	136 (86%)	79 (47%)	0 (0%)	16 (31%)
Medulloblastoma	1 (0.8%)	4 (4%)	4/120 (3%)	3 (2%)	3 (33%)	0
Meningioma	1 (0.8%)	2/42 (5%)		0	2 (22%)	2 (1.6%)
Ovarian fibroma	9/63 (14%)	9/52 (17%)	5/40 (12%)	4 (6%)	3 (43%)	4 (15%)
Cardiac fibroma			2/95 (2%)	2 (1%)	0	0

BCC Basal cell carcinomas. The definition varied according to authors: all BCC for Kimosis and Endo, > 10 BCC for Evans, multiple or before age 20 for Shanley

*KCOT* Keratocystic odontogenic tumors

Analysis of the risk of each tumor type according to the genotype is still preliminary due to the rarity of this syndrome and bias of ascertainment, since most patients belonging to Gorlin cohorts have been identified after the occurrence of multiple odontogenic keratocysts or BCCs, whereas most *SUFU* variants described so far were identified in young children with medulloblastoma.

### Basal cell carcinoma (BCC)

GS is characterized by multiple BCCs with a median age-at-onset of the first BCC of 33y (95% CI 28–37) [8]. In a large series from Manchester of 202 patients with Gorlin syndrome, the cumulative incidence of BCC was 13% in males and 12% in females by age 20, 76,5% in females and 80% in males by age 50 [8]. The risk of BCC seems to be lower in African Americans and in Japanese than in Caucasian patients from America or Europe [9, 10]. The risk of BCC seems to be greatly increased by irradiation [9]. There is some evidence for modifier genes especially the ‘red hair’ *MC1R* variant polymorphism [11] which supports some familial clustering of features beyond the *PTCH1* variant.

The risk of BCC in adult patients with germline *SUFU* mutations has not been estimated yet. Several *SUFU* PV carriers identified in GS cohorts have been diagnosed with multiple BCC [12–15], but most *SUFU* PVs were identified in infants treated for a medulloblastoma who were young at the time of clinical report and therefore their risk of BCC in adulthood cannot be assessed. In their relatives, the risk of BCC in adults is much lower than in classical GS related to *PTCH1* PVs [6] suggesting that the risk of BCC in *SUFU* PV carriers is much lower than in *PTCH1* PV carriers. Indeed, in a series of 22 *SUFU* PV carriers ascertained through a medulloblastoma, only one among 34 relatives (median age at last follow-up 51.3 years) carrying a *SUFU* PV had been diagnosed with a BCC [6].

### Odontogenic keratocysts (Jaw cysts)

Odontogenic keratocysts, are benign neoplasms of odontogenic origin. They have been described in 75–89% of patients with GS and may be the first sign of GS. These tumors seem to be restricted to *PTCH1* associated GS [4] whereas patients with *SUFU* PV are probably spared from this risk. In most series, first odontogenic keratocysts are diagnosed during the second decade of life, but jaw cysts have been diagnosed in young children during the first years of life. Even though these tumors grow slowly, their early detection is important in order to limit the impact of surgery on the growth and development of the maxillofacial complex.

### Medulloblastomas

Medulloblastoma with desmoplastic or extensive nodularity histology is the main GS-associated pediatric malignant tumor. Most tumors described so far occurred before the age of 3 years. In addition, in the context of *SUFU* PVs, families with several children affected by medulloblastomas have been described [6, 16]. In a large series of medulloblastomas screened for germline mutations, all *SUFU* or *PTCH1* PVs were observed exclusively in the SHH-medulloblastoma (SHH-MB) subgroup, with a frequency of 17/80 (21%) and 18/170 (11%) in infant and pediatric SHH MB, respectively [17].

There are currently no direct estimates of the incidence of *SUFU* and *PTCH1* PV carriers in the general population and no unbiased estimates of the risk of childhood medulloblastoma for each condition. The great majority of testing of *SUFU* is for childhood medulloblastoma and only limited testing has been carried out to identify healthy carriers in those families [17–19]. In the largest study thus far, Waszak et al. in 2018 [17], found a germline *SUFU* or *PTCH1* PV in 20/1022 (2%) patients with medulloblastoma (9 *PTCH1, 11 SUFU*). However, just over 200 of the Waszak series were over 18 years of age (Table 3), thus 11/800 (1.4%) of childhood medulloblastoma had a *SUFU* PV and 9/800 (1.1%) had a *PTCH1* PV. Brugieres et al. in 2012 found a much higher incidence of *SUFU* PVs, 8/131 (6.1%), [18] whereas Wang et al. found only one PV each for *SUFU* and *PTCH1* in their series of 129 medulloblastomas (0.8%) [19]. These differences may be related to the distribution of age-at-diagnosis and histological subtypes in the different series. Taking combined data from the three series 20/1060 (1.9%) had a *SUFU* PV and 10/929 (1.1%) had a *PTCH1* PV.

**Table 3:** Studies estimating the proportion of childhood medulloblastoma caused by *SUFU* or *PTCH1* germline variants

	Number of patients with MB tested	*SUFU*	*PTCH1*
Brugières [2]	131	8 (6.1%)	
Waszak childhood only [1]	800	11 (1.3%)	9 (1.1%)
Wang [3]	129	1 (0.7%)	1 (0.7%)
Total	1060	20 (1.8%)	10 (0.9%)

A direct estimate of medulloblastoma risk in childhood can be derived from a population-based study in the Manchester region of NW England from 1954 to 1989. In 36 years of assessment, 173 childhood medulloblastomas were registered in the Manchester Childhood tumors registry [20]. This gives an annual incidence of 4.8 in an average childhood population of 800,000 or 0.6/100,000 in the Manchester region of NW England. The childhood risk of medulloblastoma would therefore be 16 (16 years of childhood risk) × 0.6 per 100,000 = 9.6 per 100,000 or 1 in 10,400. Three patients of that series of 173 (1.73%) have subsequently been found to have a germline *SUFU* PV with two probable *PTCH1* PVs (1.15%) [13, 21]. As such, the likelihood of a childhood *SUFU* related medulloblastoma is 9.6 × 0.0173 = 0.166 per 100,000 or 1 in 601,400. The population frequency of *SUFU* PVs can be estimated from gnomAD (https://gnomad.broadinstitute.org/). Only three clearly pathogenic variants (excluding truncating variants in the last exon) were found in an average population sample of 124,185 people (1 in 41,395), although one was African and the other Finnish. Therefore the population estimate for European (non-Finnish) was 1 in 55,400. Taking these two estimates together, this would mean an estimated penetrance for medulloblastoma associated with a *SUFU* variant of 7–9.2%. Likewise, the estimate for medulloblastoma associated with *PTCH1* from gnomAD was 1 in 3356, giving an estimated penetrance of 0.37%. These estimates are about 1/3rd of those from estimated risks in Gorlin syndrome families [13]. An alternative indirect estimate based on an aggregate of around 2% for *SUFU* PVs in childhood medulloblastoma and 1% for *PTCH1* PVs from Table 3 is shown in Table 4. This generates almost identical estimates for each gene.

**Table 4:** Alternative estimate of *SUFU* and *PTCH1* based on indirect estimates from UK population incidence

Children in UK (0–16 years)	11,759,000
Annual brain tumour incidence in children	400
Incidence annual (1 in x)	29,397.5
Incidence during childhood (1 in x)	1837.3
Medulloblastoma in childhood 20% (1 in x)	9186.72
Medulloblastoma with germline *SUFU* PV (1 in x)^a^	45,9335.94
% *SUFU* PV with medulloblastoma	9.01%
Medulloblastoma with germline *PTCH1* PV (1 in x)	91,8671.9
% *PTCH* PV with medulloblastoma	0.37%

^a^Based on gnomAD 1 in 41

Considering the high incidence of *PTCH1* and *SUFU* PVs in medulloblastomas, and in particular SHH-MBs in young children, experts recommend a germline *PTCH1* and *SUFU* PVs screening for all children with SHH-MB, especially those diagnosed before the age of 5. The identification of a predisposition syndrome for medulloblastoma caused by a germline PV in *SUFU* or *PTCH1* gene is fundamental, because the presence of an underlying genetic anomaly can have an impact on the therapeutic management, due to the different behavior of *SUFU* or *PTCH1*-related medulloblastomas, including differences in their responses to SHH inhibitors [22]. The best therapeutic strategy for this group of patients still has to be assessed. In addition, identifying *SUFU* PVs in these young patients may have a major impact on familial genetic counseling and surveillance of young relatives carrying the PV, especially siblings [16].

### Meningiomas

Meningiomas have been reported in 0.8–5% of people in large series’ of GS [4, 9, 23, 24]. Meningiomas are probably more frequent in *SUFU* related GS than in *PTCH1* related GS. Indeed, in the Manchester cohort, the incidence of meningiomas was much higher in *SUFU* PV carriers (4/9) than in patients with a *PTCH1* PV (2/126). Meningiomas have also been described, mostly as case reports, in patients with *SUFU* variants, either as the first brain tumor [12, 14, 25] or, more frequently, after irradiation for a medulloblastoma [6, 13, 26, 27]. Most de novo meningiomas described so far occurred after the age of 40, whereas they occurred earlier in patients previously treated for a medulloblastoma. In addition, two multigenerational families with meningiomas as the major feature in multiple affected family members have been associated with an inherited germline *SUFU* variant [12, 25].

### Ovarian fibromas or fibrothecomas

Ovarian fibromas or fibrothecomas are benign sex-cord stromal tumors which have been described in 6–43% of female patients with GS. They are usually revealed by abdominal discomfort due to compression of adjacent organs when they become large or by acute pain due to adnexal torsion. Their incidence is probably underestimated, since most tumors are asymptomatic. With systematic screening by pelvic ultrasound, they have been detected in 9/52 (17%) females with de GS in the NIH cohort [9] and 10% in the Manchester cohort [4]. The main characteristics of GS-associated ovarian fibroma are their bilaterality and the presence of calcification. Most sporadic tumors are diagnosed in adults, but ovarian fibroma has been described in prepubertal girls in GS [28, 29]. Data from the Manchester cohort suggest that ovarian fibroma could be more frequent in *SUFU* PV female carriers (3/7) than in *PTCH1* related GS (4/68) or in patients in whom no PV was identified (4/27). Early diagnosis is important to allow detection of small tumors, resectable by minimal-access surgery.

### Cardiac fibroma

Cardiac fibroma is another hallmark tumor of GS. These tumors have been described in 1–3% of patients in large GS series [1, 10]. Most patients are diagnosed in utero or during the first years of life, but diagnoses later in life up to 60 years have been reported [30].

### Other tumors

A wide spectrum of other tumors has been reported, such as sarcomas, including rhabdomyosarcomas [6, 31–33] and thyroid carcinomas [6, 10, 34]. Due to their extreme rarity, the relative risk of these tumors in patients with GS has not been established. In addition, multiple tumor types, including sarcomas and Wilms tumors, have been described in patients with GS due to 9q22.3 microdeletion encompassing the *PTCH1* locus [35, 36].

## Recommendations for screening

Several publications including recommendation for cancer surveillance in GS have already been published [37, 38]. However, since data on tumor risks according to the gene involved were not published at that time, the European group thought it was useful to redefine surveillance guidelines adapted to the phenotype. The WHO criteria for cancer surveillance programs [39] have been taken into consideration when discussing these recommendations, even though the rarity of this condition did not allow provision of any evidence of the effectiveness of the strategy proposed, especially for pediatric tumors.

Considering the difference of incidence of each tumor type in *PTCH1* and *SUFU* PV carriers, the participants felt that screening should be adapted to the genetic background (*PTCH1-* or *SUFU*-associated GS) (Table 5). The HGWG group propose expert recommendations (not of proven evidence) that are similar to those previously published for GS associated with *PTCH1* PV, but the updated analysis of tumor risk associated with *SUFU* PV led to refined recommendations for screening for these patients. In all cases, patients should be followed annually by a medical geneticist or pediatric/adult oncologist familiar with the syndrome to check for non-tumoral manifestation of the syndromes, educate on alarming symptoms and to ensure that all screening procedures have been performed. In addition, patients/parents and their primary physician should be informed of the tumor risks associated with the syndrome, so that adequate investigation can be performed urgently in case of symptoms.

**Table 5:** Screening recommendations for patients with GS

Tumors	Methods for screening	Indications for *PTCH1* variants carriers	Indications for *SUFU* variants carriers
BCC	Dermatologic examination	Annually beginning at 10 Earlier if previous radiotherapy	Annually beginning at 20 Earlier if previous radiotherapy
KCOT	Dental examination Orthopanthogram (consider MRI)	Annually beginning at 2 Annually beginning at 8	
Medulloblastomas	Brain MRI (without contrast agent) The first can be performed with a contrast agent	Neurological examination. Brain MRI, only if symptoms or neurological signs appear	Every 3–4 months during the first 3 years then every 6 months until 5 years
Meningioma	Brain MRI		Every 3–5 years beginning at age 30 for patients with no previous MB and after healing of the MB in other patients
Ovarian tumors	Pelvic ultrasound	Once at the end of adolescence (18 years)	Every 3 years beginning at age 5 years
Cardiac fibroma	Echocardiogram	At the time of diagnosis of GS, ideally in the first 6 months of life	At the time of diagnosis of GS, ideally in the first 6 months of life

### Basal cell carcinomas

Even though the risk of BCC is probably lower in *SUFU*-related GS than in *PTCH1*-related GS, a yearly skin examination by an experienced dermatologist should be offered in both groups of patients. The age of starting should be 10 years for *PTCH1*-PV carriers and 20 years for patients with *SUFU* PVs. In both groups of patients, dermatological examination should begin earlier in people who have had previous radiotherapy in the first years after treatment completion. The interval between dermatological examinations should be shortened after the occurrence of the first BCC. In addition, recommendations for sun protection should be given in all patients.

### Odontogenic keratocysts

Early diagnosis of these benign tumors is important to facilitate a conservative local treatment. Current recommendations, which combine yearly clinical examination to follow dental eruption beginning around age 2 and annual orthopanthogram using digital imaging starting at age 8, or sooner in cases of late dental eruption, have been limited to *PTCH1* PV carriers. To avoid irradiation linked to X-rays in these patients, the use of MRI would be preferred when as sensitive as X-rays whenever possible.

Reducing imaging to once every 2 years when no cysts are observed and once every 3 years from age 30, may be suggested for long-term follow-up of these patients. As no jaw cysts have been described so far for *SUFU*-related GS, such screening is not necessary.

### Medulloblastomas

The impact of early detection of medulloblastoma on the outcome of these tumors has not been demonstrated thus far. However, theoretically, early detection should allow diagnosis of small tumors before the occurrence of metastases and easier resection of these tumors. This theoretical advantage has to be balanced against the risk associated with sedation required for good quality MRI in young children. The risk of medulloblastoma is clearly different according to the genetic background. In patients with *PTCH1* PVs in whom the risk of medulloblastoma is probably under 2%, the need for screening for medulloblastoma is not obvious. However, parents must be informed of the oncological risks and these children should be followed clinically with neurological examinations and with a high index of suspicion during the first years of life, leading to prompt brain MRI, if symptoms or neurological signs appear.

In patients with germline *SUFU* PVs, in whom the risk of medulloblastoma is estimated to be around 8–9%, brain MRIs should be recommended during the first 5 years of life. Due to rapid progression of these tumors, brain MRI should be repeated every 3 to 4 months during the first 3 years, since most tumors occur before age 3, and then every 6 months until age 5. Brain MRIs for tumor detection in healthy infant carriers are recommended without contrast agent, except for the first one which can be performed with a contrast agent. If a lesion is suspected, an additional MRI with contrast agent is necessary.

After treatment of medulloblastoma, follow-up should be prolonged and adapted to the risk of secondary tumors, such as meningioma, but also BCC and thyroid carcinomas if the patient has been treated with radiotherapy.

### Meningiomas

The benefit and harms of screening for asymptomatic meningioma is still a matter of debate especially since there are uncertainties about the most appropriate care for those tumors detected through systematic screening [40].

Due to the low incidence of meningioma in *PTCH1* PV carriers, specific screening procedures for this tumor are not required. In *SUFU* PV carriers in whom the incidence is much higher, brain MRI every 3–5 years could be proposed, beginning at age 30 years for patients with no previous medulloblastoma. For those previously treated with craniospinal irradiation, MRI of brain (+ / − spine) should be performed every 3–5 years starting after completion of medulloblastoma imaging follow-up.

### Ovarian fibroma/ fibrothecomas

Although these tumors are benign, an early detection is important to facilitate conservative surgery which may be complicated due to the high incidence of bilateral and multifocal tumors. Patients and parents should be informed of the symptoms and especially of risk of torsion so that in case of abdominal pain, imaging evaluation can be performed urgently. For females with GS associated with a *PTCH1* PV in whom the risk is probably around 5%, we recommend at least one pelvic ultrasound performed at the end of adolescence (18y). In patients with a *SUFU* PV, a first pelvic ultrasound is recommended at 10 years of age and subsequent follow-up depends on the findings. Pelvic ultrasound every 3 years is recommended if the first was normal. Monitoring should be more frequent in case of detection of a suspicious lesion. In patients in whom ovarian evaluation by ultrasound is not feasible, pelvic MRI should be discussed.

### Cardiac tumors

A baseline cardiac ultrasound should be performed at the time of diagnosis of GS, ideally within the first 6 months of life.

## Psychological and ethical issues

Like other predisposition syndromes, germline analysis for *SUFU* or *PTCH1* PV raises psychological and ethical issues. Genetic counseling is proposed to families when phenotypic criteria are present, but it is important to keep in mind that genetic testing remains offered to families, who might be willing to know but others can also prefer “not to know” which is a important right to defend. Finding such information can be stressful for children and their parents, and psychological support has to be proposed. Families should also be offered time to think about genetic issues before launching genetic tests: indeed, finding a predisposition syndrome involves lifelong monitoring, that may be difficult to live with. It also involves the possibility of genetic counseling for other family members, who may also be at risk, and communication about this risk can be difficult within some families. This is why genetic teams should include psychologists, who can help children and their parents faced with such tests to anticipate the consequences. Compliance with the legal aspect of analyses is ensured by signing the consents to perform genetic analyses. As far as possible, children, even young ones, should be involved in the process of their genetic research, with information and precautions appropriate to their age.

## Conclusion

GS was described many years ago, but the identification of different underlying molecular genetic characteristics (*PTCH1* or *SUFU* germline variants) has changed the way in which this predisposing syndrome is identified and has modified the phenotypic characteristics of the tumor risks historically described. There are still many uncertainties about the risk of tumors associated with GS. Given the rarity of this syndrome, the estimation of the risks is still not very precise and must be improved by studies on a larger number of patients with analysis taking into account ascertainment bias. Nevertheless, due to the risk of tumor, any patient with GS predisposition syndrome should benefit from a specific genotype-based surveillance program as described in these recommendations. The evaluation of the feasibility and efficacy of these recommendations is necessary.
